# High Anti-ADAMTS13 IgG Levels after Plasma Exchange Predict Delayed ADAMTS13 Normalization in Immune-Mediated Thrombotic Thrombocytopenic Purpura

**DOI:** 10.1055/a-2685-8118

**Published:** 2025-09-02

**Authors:** Marienn Réti, Andreea-Adela Icleanu, Andrea Várkonyi, Ágnes Király, Luca Bogsch, Zita Farkas, Péter Reményi, Zoltán Prohászka, György Sinkovits

**Affiliations:** 1 Department of Hematology and Stem Cell Transplantation, Central Hospital of Southern Pest - Institute of Hematology and Infectious Diseases, Budapest, Hungary; 2 Department of Internal Medicine and Hematology, Semmelweis University, Budapest, Hungary; 3 Research Group for Immunology and Hematology, Semmelweis University - Hungarian Research Network (Office for Supported Research Groups), Budapest, Hungary

**Keywords:** ADAMTS13, autoantibodies, caplacizumab, therapeutic plasma exchange, thrombotic thrombocytopenic purpura

## Abstract

**Background:**

In acute immune-mediated TTP (iTTP) caplacizumab therapy has proved to be effective in achieving an early clinical response. However, the discontinuation of caplacizumab therapy before ADAMTS13 activity has at least partially recovered can potentially lead to disease recurrence. Of note, normalization of ADAMTS13 activity was reported to be delayed in caplacizumab-treated patients.

**Aims:**

To investigate delayed ADAMTS13 normalization and its potential causes.

**Patients/Methods:**

We conducted a retrospective detailed longitudinal investigation of ADAMTS13 activity and anti-ADAMTS13 IgG levels in a single-center cohort of caplacizumab-treated iTTP patients ( *n* = 10). Results were compared to iTTP patients treated according to the standard of care in the same center, without caplacizumab (historical controls, *n* = 28).

**Results:**

We observed that ADAMTS13 activity was lower in caplacizumab-treated patients than in historical controls 1 week after therapeutic plasma exchange (TPE) was discontinued upon first clinical response (post-TPE). The difference later gradually decreased and we observed no delay in attaining ADAMTS13 activity thresholds of 20% (partial ADAMTS13 remission, reached in median 26 vs. 25 days after the first TPE session) or higher. However, almost half of the caplacizumab-treated patients needed more than 30 days to achieve partial ADAMTS13 remission. Importantly, we found that the post-TPE anti-ADAMTS13 IgG level correlates with the time until partial ADAMTS13 remission both in caplacizumab-treated and historical control patients, and is a significant predictor of delayed ADAMTS13 normalization.

**Conclusion:**

The latter finding has important clinical implications, as it suggests that measuring post-TPE anti-ADAMTS13 IgG levels may help identify patients who need additional immunosuppressive treatment to avoid delayed ADAMTS13 normalization.

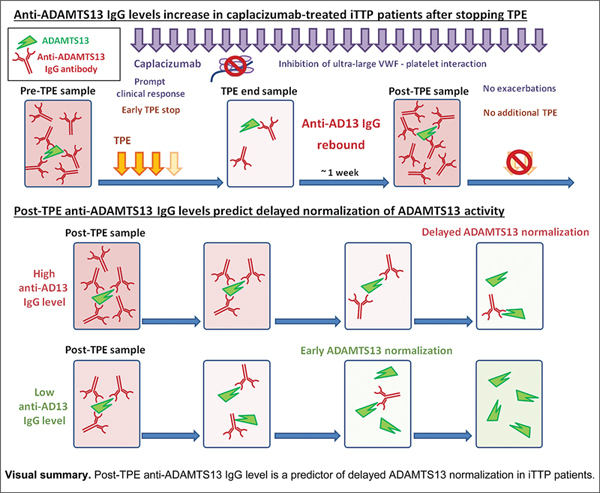

## Introduction

Immune-mediated thrombotic thrombocytopenic purpura (iTTP) is a rare disease which manifests as potentially fatal thrombotic microangiopathic episodes characterized by severe thrombocytopenia, microangiopathic hemolytic anemia, and end-organ dysfunction, caused by microvascular thrombosis. The microvascular thrombi are primarily formed by platelets that adhere to ultra-large von Willebrand factor multimers (ULVWF). 1 2 These multimers are able to bind platelets even under physiological flow conditions. 3 Therefore, under normal circumstances, they are rapidly cleaved upon secretion by the metalloprotease ADAMTS13. 4 In iTTP, however, autoantibodies are formed against the ADAMTS13 enzyme, 5 some of which are able to inactivate the enzyme directly (inhibitors), 6 whereas most antibodies also promote the clearance of the enzyme from the circulation, 7 8 leading to decreased ADAMTS13 activity and consequentially to the accumulation of the platelet-adhesive ULVWF multimers. Although anti-ADAMTS13 antibodies of the IgM and IgA isotype were described in a small proportion of iTTP cases, most anti-ADAMTS13 antibodies belong to the IgG isotype. 9 10 11

The detection of these anti-ADAMTS13 antibodies—as functional inhibitors or anti-ADAMTS13 IgG—together with the confirmation of deficient ADAMTS13 activity (usually defined as below 10% of the activity in pooled normal plasma) is an important tool in establishing the diagnosis of iTTP. 12 Moreover, the removal of anti-ADAMTS13 antibodies and the restoration of ADAMTS13 activity are central in the therapy of iTTP. According to the current standard of care, this is achieved by daily therapeutic plasma exchange (TPE) with replacement by fresh frozen plasma (FFP), and immunosuppression with corticosteroids with or without rituximab. 13 However, even with this treatment regimen, the partial restoration of ADAMTS13 enzyme activity can often be achieved only after multiple days, until which the ULVWF multimers persist, sustaining the thrombotic microangiopathy.

Caplacizumab, a therapeutic nanobody against the A1 domain of von Willebrand factor, is able to inhibit the formation of microthrombi even in the presence of ULVWF multimers, by blocking the interaction between these macromolecules and the GPIb-IX-V receptor of platelets. 14 This leads to an earlier clinical response, as shown by results of the clinical trials TITAN and HERCULES. 15 16 17 However, discontinuing caplacizumab therapy before the ADAMTS13 activity is at least partially restored confers the risk of a recurrence of the thrombotic microangiopathy due to the continued presence and formation of ULVWF multimers. 15 16

Of note, delayed normalization of ADAMTS13 activity was reported in a large cohort of caplacizumab-treated iTTP patients by Prasannan et al, 18 without an obvious explanation for the observation. This delay was later supported by results of the study by Saito et al, 19 whereas it was not observed by other groups. 20 21 22 We wanted to investigate the delayed normalization of ADAMTS13 activity and its possible causes, including its potential association with anti-ADAMTS13 antibody levels. Therefore, we conducted a detailed longitudinal investigation of ADAMTS13 activity, ADAMTS13 inhibition, and anti-ADAMTS13 IgG levels in a cohort of caplacizumab-treated iTTP patients and compared these results to those of non-caplacizumab-treated iTTP patients at specific time points.

Our observations provide additional tools that can be used in the early identification and treatment of caplacizumab-treated iTTP cases that would otherwise have a delayed normalization of ADAMTS13 activity.

## Methods

### Patient and Sample Selection

Into the caplacizumab group, we included the 10 patients who were treated with caplacizumab—in addition to the standard-of-care treatment of TPE and immunosuppression—due to an acute episode of iTTP in the Szent László Hospital Campus (Central Hospital of Southern Pest, National Institute of Hematology and Infectious Diseases, Budapest, Hungary), by the end of November 2023. Caplacizumab was included in the treatment regimen of all acute iTTP episodes from May 2021, whenever it was available. The historical control group consisted of 28 patients who were treated at our center between January 2017 and November 2023 for an acute iTTP episode according to the standard of care, but without caplacizumab. The criteria and steps of patient selection are summarized in Supplementary Fig. S1 (available in the online version).

**Fig. 1:**
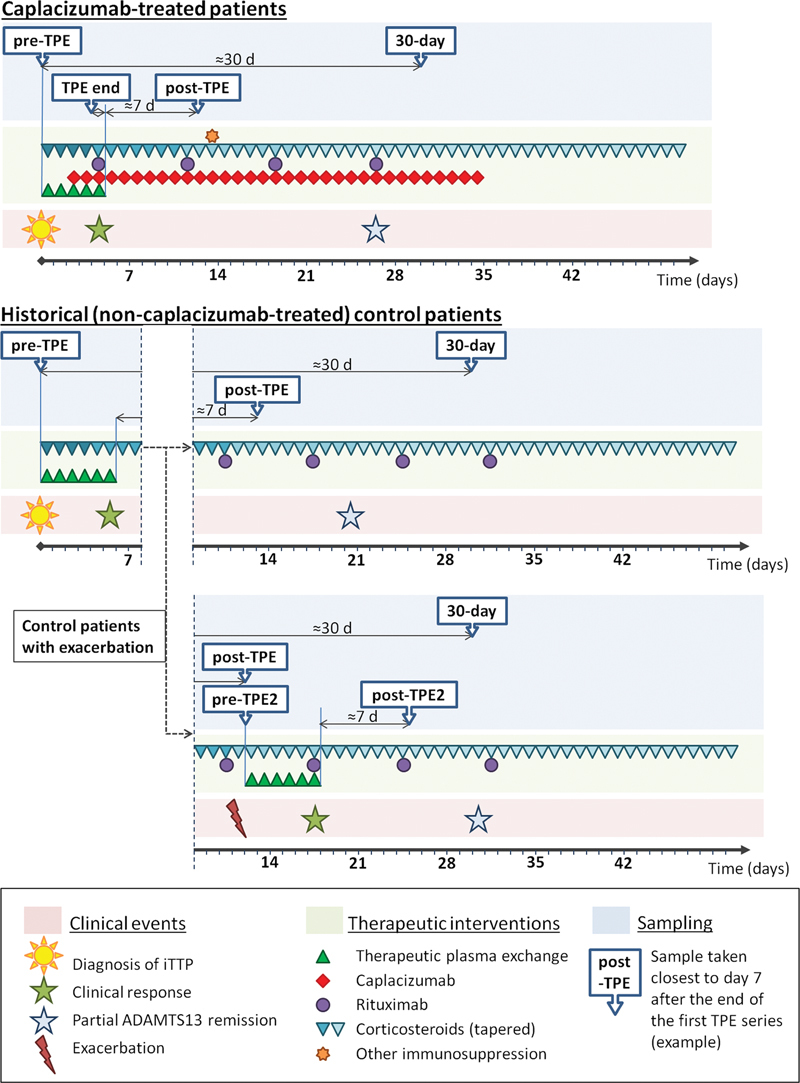
Overview of the characteristic clinical course of caplacizumab-treated and historical control immune-mediated thrombotic thrombocytopenic purpura (iTTP) patients. Time points of clinical events, therapeutic interventions, and sampling times are plotted based on the median time points in the respective groups: caplacizumab-treated patients, non-exacerbating and exacerbating historical control patients. Samples were not taken in a uniform manner, but based on the patient's clinical state, initially generally at least weekly. Samples closest to the key time points were chosen for certain analyses, if they were available, such as the sample taken before starting therapeutic plasma exchange (TPE) (pre-TPE), 1 week after the end of the first TPE series (post-TPE), and 30 days after the first TPE session (30-day). Post-TPE samples were taken before the re-initiation of TPE in exacerbating control patients. In addition to the above time points, eight caplacizumab-treated patients had samples taken at the end of TPE, that is, in the last 2 days before stopping the TPE series for seven patients and 1 day after stopping TPE for one patient (TPE end). Clinical response is defined as platelet count above 150 × 10 ^9^ /L without signs of hemolysis for at least 2 days, allowing the discontinuation of TPE. Sustained partial ADAMTS13 remission is defined as the first day of ADAMTS13 activity over 20% for at least 30 days (allowing single low values not lower than 17% [85% of the threshold] and starting at least 2 days after the last TPE session). Exacerbation is considered if the platelet count decreases to <150 × 10 ^9^ /L (other causes of thrombocytopenia excluded) after an initial clinical response, with deficient ADAMTS13 activity, due to which TPE has to be restarted.

Blood samples for ADAMTS13 (activity, inhibitor, and IgG antibody) determination were taken by venipuncture or from a central venous catheter. Tubes were transported in cooled packages (4°C) in less than 4 hours to the TMA research laboratory (Research Laboratory, Department of Internal Medicine and Hematology, Semmelweis University, Budapest, Hungary) where they were centrifuged. Separated serum, citrated and EDTA-anticoagulated plasma aliquots were stored at −70°C until measurements. For caplacizumab-treated patients, we aimed to perform ADAMTS13 measurements at least weekly from the day of admission to our center (before the first TPE) until the normalization of the ADAMTS13 activity. Key sampling times in relation to clinical course and therapy are summarized in Fig. 1 .

The present study was conducted in accordance with the Declaration of Helsinki, written informed consent was obtained from all participating patients, and the study was approved by the Human Research Ethics Committee of the Hungarian Medical Research Council (8361-1/2011-EKU(263/PI/11)). Sanofi's Access Program provided caplacizumab vials in half of the cases.

### Treatment Regimen, Protocols, Outcome Definitions

The treatment regimen of iTTP patients at our center is described in detail in the Supplementary Material (available in the online version).

Caplacizumab was applied according to the drug's instructions for use. The first 10 mg dose of caplacizumab was intravenously given before plasma exchange, followed by 10 mg subcutaneous injections daily (given immediately after each plasma exchange) and continued daily for 30 days after discontinuation of daily TPE sessions. Treatment could be extended for patients whose ADAMTS13 activity had not yet normalized, and was suspended for patients not enrolled in Sanofi's Access Program after their discharge from the hospital.

The outcome definitions were used in line with the 2021 consensus report of the International Working Group for TTP with some amendments. 23 Initial platelet response was defined as platelet count above 150 × 10 ^9^ /L for the first time. Clinical response was defined as platelet count above 150 × 10 ^9^ /L without signs of hemolysis for at least 2 days, allowing the discontinuation of TPE. Exacerbation was diagnosed if the platelet count decreased below 150 × 10 ^9^ /L (with other causes of thrombocytopenia excluded) after an initial clinical response, with deficient ADAMTS13 activity, due to which TPE had to be restarted. Permanent platelet response was defined as the first day of an at least 30-day period with platelet count above 150 × 10 ^9^ /L. Regarding the (partial or complete) normalization of ADAMTS13, we considered the first sample fulfilling the above criteria: (1) ADAMTS13 activity above the threshold, (2) sample taken at least 2 days after the last TPE, (3) ADAMTS13 activity remained above the threshold for at least 30 days afterwards, allowing isolated low values not lower than 85% of the threshold. Sustained partial ADAMTS13 remission was established when the ADAMTS13 activity exceeded the threshold of 20% according to the above criteria. ADAMTS13 normalization was considered delayed if sustained partial ADAMTS13 remission was achieved in more than 30 days from the first TPE session (indicating the start of iTTP-specific therapy). Remission was defined as sustained clinical response with no TPE and no anti-VWF therapy for ≥30 days or until the first day of ADAMTS13 activity permanently above 20%, whichever occurred first.

### Laboratory Determinations

ADAMTS13 activity was determined in citrated plasma samples by a FRETS-VWF73 assay. 24

The presence and strength of functional anti-ADAMTS13 inhibition were assessed in patient samples with deficient or near-deficient (≤15%) ADAMTS13 activity, by measuring the ADAMTS13 activity of a 1:1 mixture of the ADAMTS13-deficient citrated plasma sample and normal, pooled human citrated plasma (with a nominal ADAMTS13 activity of 100%) following 2 hours of incubation at 37°C. The activity of the mixed sample was normalized to that of incubated normal human citrated plasma (regarded as 100%). The normalized activity of a mixed sample would theoretically be 50% in the absence of detectable inhibitors, with lower activities indicating a stronger inhibition. For samples with an ADAMTS13 activity above 15% (where no additional inhibition is expected), the measurement was not performed; missing data were replaced by the average of the sample's ADAMTS13 activity and that of the normal pooled sample (100%).

Anti-ADAMTS13 IgG levels were measured in serum or citrated plasma samples by a commercial ELISA kit (Technozym ADAMTS13 Inh; Technoclone GmbH, Vienna, Austria). If citrated plasma samples were measured, results were divided by 0.9 to correct for sample dilution with citrate. Anti-ADAMTS13 IgG results above 15 U/mL were regarded positive, following the manufacturer's recommendation.

### Statistical Analysis

As the distributions of most variables were not normal, median and quartiles were used for the description of data (if not otherwise specified) and nonparametric statistical tests were used. The Mann-Whitney test was used to compare continuous variables between two unrelated groups. Proportions were compared by Fisher's exact test. Bonferroni's method was used to correct for multiple comparisons.

Statistical calculations were performed by the GraphPad Prism 9 software (GraphPad Softwares Inc., La Jolla, CA, USA). Logistic regression models were used to assess potential predictors of delayed ADAMTS13 normalization; these analyses were performed by the Statistica software (version 13.5.0.17, TIBCO Software Inc., Palo Alto, California, United States).

## Results

### Description of the Patient Cohorts

Demographic and clinical characteristics of the caplacizumab-treated and historical control patients are presented in Table 1 .

**Table 1:** Demographic and clinical characteristics of caplacizumab-treated and control iTTP patients

	Caplacizumab ( *n* = 10)	Control ( *n* = 28)	*p* -value
Age (years; median, range)	39 (23–57)	42 (18–69)	0.4666
Male sex, % ( *n* )	40.0 (4)	21.4 (6)	0.4036
First acute episode, % ( *n* )	70.0 (7)	53.6 (15)	0.4694
ADAMTS13 activity pre-TPE (%; median, range)	0 (0–0)	0 (0–6)	0.1821
ADAMTS13 inhibition pre-TPE (activity of the mixed sample, a %, median, range)	0 (0–3)	6 (0–43)	0.0151
Anti-ADAMTS13 IgG pre-TPE (IU/mL; median, range)	103.5 (43.4–139.9)	76.7 (16.3–144.8)	0.0549
Caplacizumab started (days after TPE start; median, range)	2 (0–8)	−	−
Length of caplacizumab therapy f (days; median, range)	33 (22–102)	−	−
ADAMTS13 activity when caplacizumab stopped f (%; median, range)	52 (0–74)	−	−
TPE sessions until first clinical response b (median, range)	5 (3–11)	6 (2–20)	0.1523
TPE sessions until remission c, f (median, range)	5 (3–11)	11.5 (3–21)	**0.0021**
Rituximab-treated patients, % ( *n* )	80.0 (8)	67.9 (19)	0.6900
Rituximab started (days after TPE start; median, range)	4 (3–19)	10 (–1; 31)	0.0808
Rituximab total dose (mg; median, range)	400 (300–700)	400 (200–760)	0.5140
Steroid-treated patients, % ( *n* )	100 (10)	100 (28)	1.0000
Steroid starting dose (mg; median, range)	120 (80–160)	80 (80–250)	0.6323
Patients treated with other ISU, % ( *n* ) g	40.0 (4)	10.7 (3)	0.0625
Time until initial platelet response d (days after TPE start; median, range)	4 (2–10)	4 (2–22)	0.3615
Time until initial platelet response c (days after caplacizumab start; median, range)	2 (0–3)	−	−
Time until permanent platelet response e h (days after TPE start; median, range)	6.5 (2–21)	13 (3–101)	0.1119
Exacerbation, % ( *n* ) f	0 (0)	50 (14)	0.0071
Mortality, % ( *n* ) f	0 (0)	0 (0)	1.0000

Abbreviations: ISU, immunosuppressive therapy; iTTP, immune-mediated thrombotic thrombocytopenic purpura; TPE, therapeutic plasma exchange.

Notes: The control group consists of iTTP patients treated at the same center after January 2017 according to the standard of care, but without caplacizumab.

*p* -values of the Mann-Whitney test (for continuous variables) or Fisher's exact test (for categorical variables) are shown; significant *p* -values after the Bonferroni correction (significance limit *p* = 0.0023) are indicated in bold.

a ADAMTS13 activity of a 1:1 mixture of the patient sample and a pooled normal human sample with a nominal activity of 100%, incubated for 2 hours at 37°C. Lower activities indicate stronger inhibition.

b Platelet count above 150 × 10 ^9^ /L for at least 2 days, allowing the discontinuation of TPE.

c Sustained clinical response with no TPE and no anti-VWF therapy for ≥30 days or until the first day of ADAMTS13 activity permanently above 20%, whichever occurs first.

d Platelet count above 150 × 10 ^9^ /L for the first time since admission.

e First day of a normal platelet count (>150 × 10 ^9^ /L) sustained for at least 30 days.

f One patient excluded because caplacizumab therapy was discontinued.

g Other immunosuppressive agents applied: cyclophosphamid, bortezomib, daratumumab.

h One patient excluded due to discontinued caplacizumab therapy and a further one excluded because of persistently low platelet levels due to other reasons (despite near-normal ADAMTS13 activity), respectively.

There was no significant difference in the age and gender distribution of caplacizumab-treated and historical control patients. More than half of the patients were included in relation to a first acute episode in both groups.

ADAMTS13 activity was similarly deficient in both groups. The strength of the ADAMTS13 inhibition and the level of anti-ADAMTS13 IgG antibodies tended to be higher in the caplacizumab-treated group, but the difference was not significant after correction for multiple comparisons.

Caplacizumab was started at a median of 2 days after initiating TPE. Initial platelet response (platelet count above 150 × 10 ^9^ /L for the first time) was reached in the first 3 days of caplacizumab treatment. Caplacizumab therapy had to be discontinued in one patient after 19 days due to the patient's decision. In the other patients, caplacizumab therapy was continued for a median of 33 days, but the length of therapy varied between 22 and 102 days. Six patients had reached and another one had almost achieved sustained partial ADAMTS13 remission (ADAMTS13 activity above 20% for at least 30 days, see the “Methods” section for details) by the time caplacizumab therapy was discontinued, whereas two patients were still ADAMTS13-deficient.

All patients were treated with corticosteroids in addition to TPE. The median number of TPE sessions until the first clinical response (platelet count above 150 × 10 ^9^ /L without signs of hemolysis for at least 2 days, allowing the discontinuation of TPE) was similar, but the historical control patients had more than twice as much TPE sessions in total than caplacizumab-treated patients, because TPE had to be restarted in half of them due to an exacerbation (platelet count drop below 150 × 10 ^9^ /L after a clinical response, requiring further TPE sessions). The proportions of patients treated with rituximab in the two groups were similar. The dosage of rituximab did not differ either, but the caplacizumab-treated patients tended to receive rituximab treatment approximately 1 week earlier. The proportion of patients treated with other immunosuppressive therapies also tended to be higher in the caplacizumab-treated group.

Interestingly, there was no difference in the median time from TPE start until initial platelet response (4 days in both groups) but it was more variable with values up to 22 days in the historical control group. The median time until the permanent platelet response (without further exacerbation) was 1 week shorter in caplacizumab-treated patients than in historical control patients, although the difference was not significant.

None of the caplacizumab-treated patients had an exacerbation requiring further TPE sessions, although three of them did have slight isolated thrombocytopenia that resolved in a few days. As mentioned earlier, TPE had to be restarted in half of the historical control patients due to an exacerbation. All patients survived the studied acute TTP episodes.

### Normalization of ADAMTS13 Activity

**Fig. 2:**
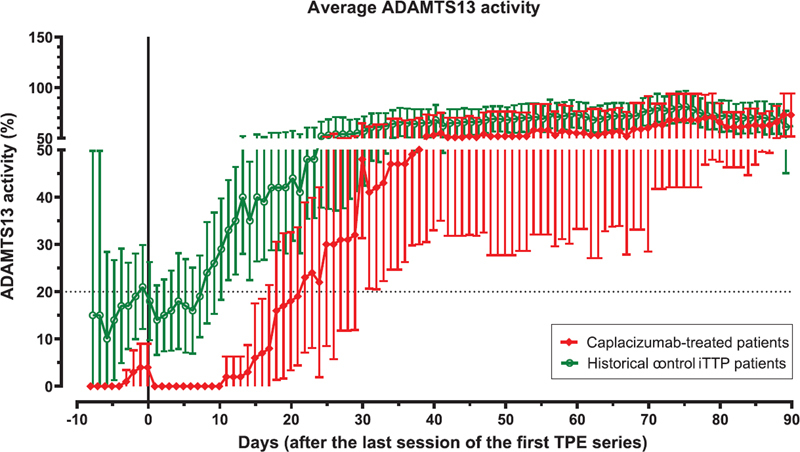
ADAMTS13 activity normalization in caplacizumab-treated and historical control immune-mediated thrombotic thrombocytopenic purpura (iTTP) patients. Mean ADAMTS13 activity values and their 95% confidence intervals are shown. The vertical line indicates the zero time point, which is the last day of the first therapeutic plasma exchange (TPE) series (TPE discontinued on the first day of clinical response). For each day's mean, one value per patient, on or closest to the index day, was included in the calculation. Samples taken more than a week (until day 30) or 2 weeks (from day 30) after the given time point were not included. The mean sampling interval in the first month of follow-up was 3.6 days in caplacizumab-treated patients and 5.2 days in the historical control patients. Results measured during TPE sessions—comprising additional TPE series initiated due to exacerbations in 14 control patients—were also included in this analysis. The horizontal dotted line indicates the threshold of partial ADAMTS13 remission (20%). Individual values of caplacizumab-treated patients are shown in Fig. 3A , whereas those of historical control patients are shown in Supplementary Fig. S2 (available in the online version).

First, we compared the recovery of ADAMTS13 activity in caplacizumab-treated and historical control iTTP patients. TPE was discontinued upon the first clinical response, and in the sample taken 1 week thereafter (post-TPE sample) we observed lower ADAMTS13 activity in caplacizumab-treated patients than in historical control iTTP patients (0 [0–0] vs. 5 [0–14] U/mL). The difference remained significant for almost 3 weeks post-TPE, but gradually disappeared thereafter ( Fig. 2 ).

The intrinsic ADAMTS13 activity—sustained for at least 30 days without subsequent TPE sessions—exceeded the threshold of deficiency (10%) 4 days later in caplacizumab-treated patients than historical control patients in relation to the first TPE session (26 vs. 22 days), although the difference was not significant. However, the median times until sustained partial ADAMTS13 remission (>20%) were already similar (26 vs. 25 days) in the two groups. There was also no difference observed at the 30, 50, and 67% thresholds ( Table 2 ).

**Table 2:** Normalization of ADAMTS13 activity and persistence of anti-ADAMTS13 IgG in caplacizumab-treated and control iTTP patients

	Caplacizumab ( *n* = 9)	Control ( *n* = 28)	*p* -value
Time to ADAMTS13 activity >10% (days; median, range)	26 (17–80)	22 (5–47)	0.0874
Time to ADAMTS13 activity >20% (days; median, range)	26 (17–87)	25 (5–179)	0.2736
Time to ADAMTS13 activity >30% (days; median, range)	28 (22–87)	29.5 (5–277)	0.3144
Time to ADAMTS13 activity >50% a (days; median, range)	44 (31–144)	39 (8–543)	0.6697
Time to ADAMTS13 activity >67% a (days; median, range)	88 (38–208)	55 (17–1,134)	0.4581
Time to anti-ADAMTS13 IgG <15 U/mL (days; median, range)	35 (19–80)	na	na
Anti-ADAMTS13 activity in the first negative sample (%; median, range)	29 (0–60)	na	na
Anti-ADAMTS13 IgG 30 days after the first TPE (U/mL; median, range)	15.8 (1.3–121.6)	5.5 (1.9–80.8)	0.0720

Abbreviations: iTTP, immune-mediated thrombotic thrombocytopenic purpura; na, data not available.

Notes: Days elapsed from the first TPE session until the first sample fulfilling the criteria were considered. For partial or complete sustained normalization of ADAMTS13 activity, the following criteria had to be met: (1) ADAMTS13 activity above the threshold, (2) sample taken at least 2 days after the last TPE, and (3) ADAMTS13 activity remained above the threshold for at least 30 days afterwards, allowing isolated low values not lower than 85% of the threshold. One caplacizumab-treated patient was excluded from these analyses due to the discontinuation of caplacizumab therapy.

*p* -values of the Mann-Whitney test (for continuous variables) or Fisher's exact test (for categorical variables) are shown.

a One control patient did not reach 50% ADAMTS13 activity whereas further three control patients and one caplacizumab-treated patient did not reach 67% activity during their entire follow-up or before another relapse. For the analyses, their results were substituted by the maximum value of the respective group.

### Associations Between Anti-ADAMTS13 IgG Levels and ADAMTS13 Activity

**Fig. 3:**
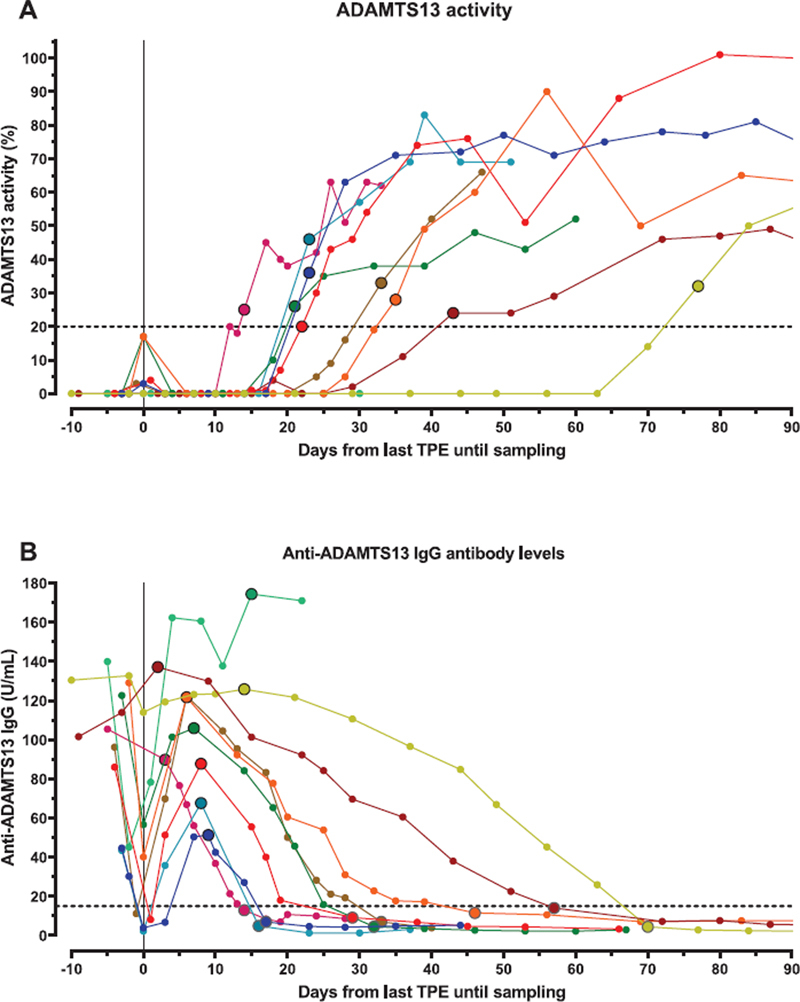
ADAMTS13 activity (A) and anti-ADAMTS13 IgG antibody levels (B) in samples of caplacizumab-treated immune-mediated thrombotic thrombocytopenic purpura (iTTP) patients. Matching colors on panels A and B refer to the same patient. Large icons indicate the first ADAMTS13 activity value that reached 20% on panel A, and the peak post–therapeutic plasma exchange (TPE) anti-ADAMTS13 IgG values (black border) and the first anti-ADAMTS13 IgG value below the diagnostic cut-off of 15 U/mL (gray border) on panel B. The horizontal dashed line indicates the cut-off values of 20% ( **A** ) and 15 U/mL ( **B** ). The vertical black line indicates the last day of TPE. Results of patient 8 (turquoise) after the discontinuation of caplacizumab therapy are excluded.

In order to assess how the anti-ADAMTS13 antibody levels change in parallel with the normalization of ADAMTS13 activity, we performed a detailed investigation of anti-ADAMTS13 IgG antibody levels in caplacizumab-treated patients ( Fig. 3 ).

All caplacizumab-treated patients had strong ADAMTS13 inhibition and markedly positive anti-ADAMTS13 IgG levels immediately before starting the TPE therapy. By the end of the TPE series, levels of these antibodies dropped significantly in most patients. However, after TPE therapy was stopped upon reaching clinical response—which occurred after a similar number of TPE sessions as in historical control patients—the level of anti-ADAMTS13 IgG antibodies and the strength of ADAMTS13 inhibition increased and ADAMTS13 activity turned deficient again in all caplacizumab-treated patients. Anti-ADAMTS13 IgG levels reached a peak approximately 1 week (8 [5–11] days) after the last TPE session. Accordingly, anti-ADAMTS13 IgG values measured 1 week after stopping the TPE therapy (post-TPE) were comparable or even higher than before TPE. The median time until ADAMTS13 IgG levels turned negative (<15 U/mL) was 35 days from the first TPE session, but antibodies persisted for up to 80 days in certain cases ( Table 2 ).

**Fig. 4:**
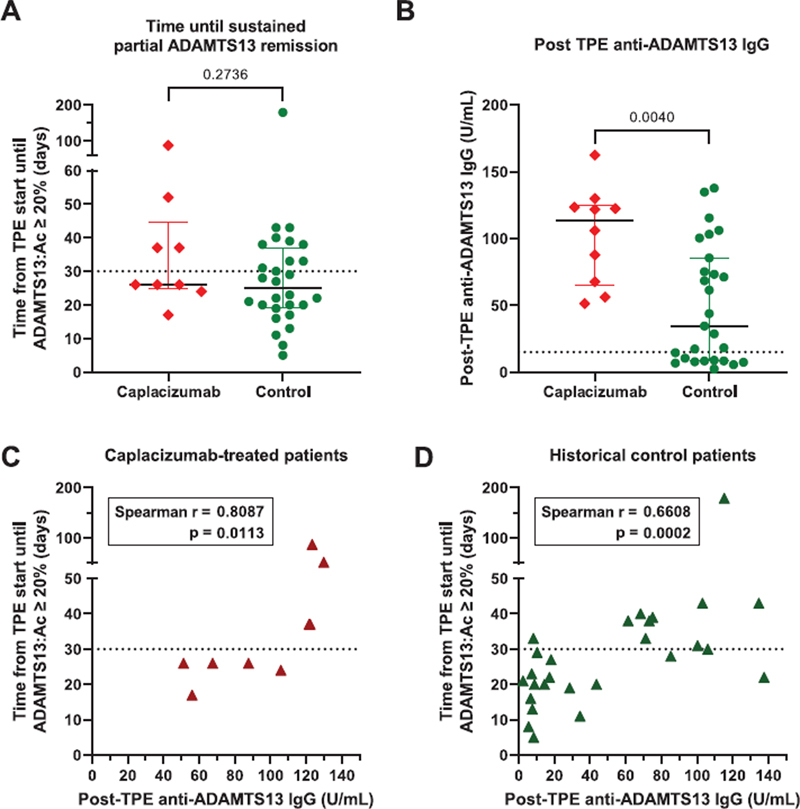
Post–therapeutic plasma exchange (TPE) anti-ADAMTS13 IgG levels and time until sustained partial ADAMTS13 remission. **(A)** Time from the first TPE series to sustained partial ADAMTS13 remission in caplacizumab-treated and historical control immune-mediated thrombotic thrombocytopenic purpura (iTTP) patients. Sustained partial ADAMTS13 remission is defined as ADAMTS13 activity above 20% for at least 30 days. **(B)** Post-TPE antibody levels in caplacizumab-treated and historical control patients. Post-TPE samples were taken approximately 1 week (6 [5–8.5] days) after the end of the first TPE series. The horizontal dashed line indicates the threshold of positivity. **(C, D)** Correlations between post-TPE anti-ADAMTS13 IgG levels and the time until sustained partial ADAMTS13 remission in caplacizumab-treated patients **(C)** and in historical control patients **(D)** . ADAMTS13 normalization is considered delayed if sustained partial ADAMTS13 remission is reached in more than 30 days from TPE start; the horizontal dashed line indicates this threshold on panels **A** , **C** , and **D** .

The prolonged persistence of antibodies in certain patients is in line with our observation that although the median time until sustained partial ADAMTS13 remission did not differ between caplacizumab-treated and historical control patients, individual values varied markedly in both groups, with a substantial proportion of patients (44% in the caplacizumab group) requiring more than 30 days to reach sustained partial ADAMTS13 remission ( Fig. 4A ). The time until sustained partial ADAMTS13 remission was strongly correlated with the time until anti-ADAMTS13 IgG negativity (Spearman r: 0.8548, *p* = 0.0049), which indicates that the prolonged persistence of these antibodies plays a role in delayed ADAMTS13 normalization.

Thus, we next investigated whether anti-ADAMTS13 IgG levels measured at specific time points are associated with the time until sustained partial ADAMTS13 remission. Importantly, we found that post-TPE anti-ADAMTS13 IgG values—but not pre-TPE antibody levels (Spearman r: 0.3065, *p* = 0.4204)—measured in caplacizumab-treated patients were not only higher than those in historical control patients ( Fig. 4B ), but were also strongly correlated with the time to sustained partial ADAMTS13 remission ( Fig. 4C ). All caplacizumab-treated patients with post-TPE anti-ADAMTS13 IgG levels above 110 to 120 U/mL ( *n* = 4) had a delayed partial ADAMTS13 normalization, whereas patients with antibody levels below that range ( *n* = 5) all reached a sustained ADAMTS13 activity of ≥20% within 30 days after the first TPE session ( Fig. 4C ). Although the time until sustained partial ADAMTS13 remission may have been influenced by additional TPE sessions in exacerbating control iTTP patients, it also correlated with post-TPE antibody levels in the historical control group ( Fig. 4D ).

Similarly, we observed that the ADAMTS13 activity of the post-TPE samples mixed and incubated together in a 1:1 ratio with normal human samples (mixed samples) was lower in caplacizumab-treated patients, indicating a stronger ADAMTS13 inhibition. The activities of the mixed post-TPE samples—that is, the strength of post-TPE ADAMTS13 inhibition—also correlated with the time to sustained partial ADAMTS13 remission both in the caplacizumab and in the historical control group (data not shown).

### Post-TPE Anti-ADAMTS13 IgG Levels as Predictors of Delayed ADAMTS13 Activity Normalization

In line with the above findings, we wanted to investigate the utility of post-TPE anti-ADAMTS13 antibody levels, and other laboratory measures and clinical factors to predict a delayed normalization of ADAMTS13 activity, defined here as reaching a sustained partial ADAMTS13 remission (activity >20% for at least 30 days) in more than 30 days from the start of TPE therapy. The most relevant potential clinical and laboratory predictors of patients with or without delayed ADAMTS13 normalization are shown in Table 3 .

**Table 3:** Comparison of iTTP patients with or without delayed ADAMTS13 normalization

	Delayed (sustained partial ADAMTS13 remission in >30 days; *n* = 14)	Non-delayed (sustained partial ADAMTS13 remission in ≤30 days; *n* = 23)	*p* -value
Age (years; median, range)	48 (18–62)	41 (18–69)	0.5942
Male sex, % ( *n* )	14.3 (2)	34.8 (8)	0.1642
Relapse, % ( *n* )	28.6 (4)	47.8 (11)	0.3136
Caplacizumab-treated patients, % ( *n* )	28.6 (4)	21.7 (5)	0.7046
TPE sessions until first clinical response a (median, range)	7 (2–15)	5 (3–20)	0.3162
TPE sessions until remission b (median, range)	10.5 (3–21)	7 (3–20)	0.1813
Rituximab-treated patients, % ( *n* )	78.6 (11)	69.6 (16)	0.7099
Rituximab started (days after TPE start; median, range)	10 (–1; 26)	5 (2–31)	0.2651
Rituximab total dose (mg; median, range)	400 (300–700)	400 (200–760)	0.6977
Steroid starting dose (mg; median, range)	80 (80–125)	125 (80–250)	0.0999
Patients treated with other ISU, c % ( *n* )	21.4 (3)	13.0 (3)	0.6534
Platelet count, pre-TPE (10 ^9^ /L; median, range)	13.5 (3–33)	13.5 (4–38)	0.9094
ADAMTS13 activity, pre-TPE (%; median, range)	0 (0–4)	0 (0–6)	0.3085
ADAMTS13 activity, post-TPE (%; median, range)	0 (0–14)	3 (0–73)	0.0728
ADAMTS13 inhibition, pre TPE (activity of the mixed sample, d %, median, range)	1.5 (0–43)	3 (0–37)	0.7530
ADAMTS13 inhibition, post TPE (activity of the mixed sample, d %, median, range)	3 (0–65)	39 (0–87)	0.0393
Anti-ADAMTS13 IgG, pre-TPE (U/mL; median, range)	97.3 (32.2–143.9)	45.0 (16.3–144.8)	0.0196
Anti-ADAMTS13 IgG, post-TPE (U/mL; median, range)	101.7 (8.3–134.7)	23.4 (2.4–137.6)	**0.0014**

Abbreviations: ISU, immunosuppressive therapy; iTTP, immune-mediated thrombotic thrombocytopenic purpura; TPE, therapeutic plasma exchange.

Notes: Delayed ADAMTS13 normalization: Sustained partial ADAMTS13 remission is achieved after more than 30 days from the first TPE session.

Sustained partial ADAMTS13 remission: ADAMTS13 activity ≥20% for at least 30 days (starting at least 2 days after the last TPE session and allowing single low values not lower than 17% (i.e., 85% of the threshold)).

*p* -values of the Mann-Whitney test (for continuous variables) or Fisher's exact test (for categorical variables) are shown; significant *p* -values after the Bonferroni correction (significance limit *p* = 0.0028) are indicated in bold.

a Platelet count above 150 × 10 ^9^ /L for at least 2 days, allowing the discontinuation of TPE.

b Sustained clinical response with no TPE and no anti-VWF therapy for ≥30 days or until the first day of ADAMTS13 activity permanently above 20%, whichever occurs first.

c Other immunosuppressive agents applied: cyclophosphamid, bortezomib, daratumumab.

d ADAMTS13 activity of a 1:1 mixture of the patient sample and a pooled normal human sample with a nominal activity of 100%, incubated for 2 hours at 37°C. Lower activities indicate stronger inhibition.

We found that pre-TPE and post-TPE anti-ADAMTS13 IgG levels were higher in patients with a delayed ADAMTS13 normalization than in the other subgroup, although only the latter difference was significant after correction for multiple comparisons. In contrast, neither ADAMTS13 activity or the strength of ADAMTS13 inhibition—expressed by the ADAMTS13 activity of the mixed sample—and none of the investigated demographic, clinical, or routine laboratory parameters differed significantly between the two groups.

Next, we performed univariable logistic regression models on the pooled data of caplacizumab-treated and historical control patients to investigate the potential of the above clinical and laboratory variables—including early rituximab administration (started during the first week from TPE start)—to predict the probability of a delayed ADAMTS13 normalization ( Supplementary Table S1 , available in the online version).

In line with the group comparison, only the post-TPE anti-ADAMTS13 IgG level proved to be a significant predictor of a delayed normalization ( *p* = 0.0030).

We determined the optimal cut-off values to distinguish between patients with delayed and non-delayed ADAMTS13 normalization. As the different TPE regimens applied in caplacizumab-treated and historical control patients may influence the associations between post-TPE levels and delayed normalization, we addressed these two groups separately ( Supplementary Fig. S3 , available in the online version). We found that the optimal cut-off value was around 110 U/mL for caplacizumab-treated and 50 U/mL for non-caplacizumab-treated iTTP patients.

Historical control patients with anti-ADAMTS13 IgG levels above 50 U/mL post-TPE have a 42 times (95% confidence interval: 3.3–530) higher risk of a delayed ADAMTS13 normalization, whereas the exact risk could not be calculated for caplacizumab-treated patients because the cut-off perfectly discriminated between patients with delayed and non-delayed ADAMTS13 normalization.

## Discussion

In this study, we performed a detailed longitudinal investigation of anti-ADAMTS13 IgG levels and ADAMTS13 activity in a cohort of caplacizumab-treated iTTP patients and compared their results to those of non-caplacizumab-treated iTTP patients (historical controls) at specific time points. The aims were to determine whether the normalization of ADAMTS13 activity is delayed in caplacizumab-treated patients and to identify factors that could predict a delayed ADAMTS13 normalization. Our results provide useful insights that may be used to tailor therapeutic modalities to fit individual cases.

We found that after discontinuing TPE therapy upon the first clinical response, the mean ADAMTS13 activity was lower in the caplacizumab group than in the historical control group for approximately 3 weeks ( Fig. 2 ). This is probably attributable to two main reasons. First, whereas 33% of control iTTP patients had an ADAMTS13 activity of at least 10% in the post-TPE sample (taken before the eventual re-initiation of TPE upon exacerbation), all of the caplacizumab-treated patients were still deficient at this time. Second, TPE had to be restarted in the majority of historical control patients with deficient post-TPE values (72%, 13/18)—thereby restoring their ADAMTS13 activity—whereas none of the caplacizumab-treated patients had an exacerbation requiring TPE again. Both of the above reasons can be traced back to the efficacy of caplacizumab in reaching a prompt clinical response—allowing for the discontinuation of TPE before the ADAMTS13 activity recovered—and in preventing clinical exacerbations later on.

In line with the initially lower ADAMTS13 activity observed in caplacizumab-treated patients, we found that the time from TPE start until the restoration of intrinsic ADAMTS13 activity (i.e., sustained without TPE) to 10% tended to be higher in this group, although the difference was not significant ( Table 2 ). However, the times until thresholds of 20% or higher were reached were no longer different, probably due to the earlier use of rituximab and the additional immunosuppression applied in selected caplacizumab-treated patients with deficient ADAMTS13 activity and high anti-ADAMTS13 IgG levels.

The median times until partial recovery of ADAMTS13 activity in our caplacizumab-treated patients were comparable to, but tended to be lower than, those observed in studies from the United Kingdom (30% in 25 vs. 31 days post-TPE), 18 France (20% in 23 vs. 28 days post-TPE), 20 Germany (10% in 26 vs. 32.5 days), 21 Spain (10% in 26 vs. 28 days), 22 and in Japan (10% in 26 vs. 42 days). 19 The shorter time to recovery may be in part due to the early additional immunosuppression guided by the frequently monitored ADAMTS13 activity and antibody results.

Of note, some of the above groups reported the time from the first TPE, whereas others—including Prasannan et al 18 —compared the times from the last TPE session. This may contribute to contrasting conclusions regarding the delayed or non-delayed normalization in caplacizumab-treated patients, as TPE was stopped earlier in caplacizumab-treated patients compared to controls in many studies.

It is worth mentioning that eight of our patients were also included in The Capla 1000+ Project, 25 in which there was also no difference observed in time to ADAMTS13 activity of >20% from the first TPE between caplacizumab-treated and control iTTP patients.

Even though the time until partial ADAMTS13 remission was not significantly longer in caplacizumab-treated patients in our study, it was indeed highly variable ( Table 2 , Fig. 4A ). Partial normalization was delayed for up to 87 days after TPE initiation in some patients, which is in line with findings of previous groups. 18 19 20 21

In order to investigate how these individual differences are related to changes in anti-ADAMTS13 antibody concentrations, we performed a detailed longitudinal investigation of anti-ADAMTS13 IgG levels in caplacizumab-treated patients.

Although TPE effectively reduced antibody levels, we observed a rebound after TPE was discontinued upon clinical response, with values higher than the baseline (pre-TPE) in the majority of patients ( Fig. 3 ). There are several potential causes of this remarkable phenomenon. A TPE session with one-and-a-half plasma volume removes approximately 70% of substances in the intravascular space, which is followed by a slow influx from the extravascular compartment. 26 Consequentially, removal of extravascular pathogenic IgG antibodies may be incomplete after a few TPE sessions, and thus a re-emergence may occur after stopping TPE even if antibody production is blocked. 27 In iTTP, however, the applied immunosuppressive agents—corticosteroids and rituximab—do not cease antibody production immediately. 28 29 As caplacizumab allows earlier discontinuation of TPE due to a prompt clinical response, the rate of antibody production may still be high at the time of TPE discontinuation, which might explain the higher prevalence of the antibody rebound in caplacizumab-treated patients.

Moreover, TPE also acts by replenishing the missing ADAMTS13 protease. Prolonged antigen stimulation by plasma replacement may help induce immune tolerance in itself, 30 31 but may also augment the immune response in the short term, via the so-called inhibitor boosting. 32 Interestingly, Kühne et al reported shorter times from caplacizumab start to ADAMTS13 remission in patients treated with caplacizumab and immunosuppression but without TPE than in those also treated with TPE, 33 which is in line with the above hypothesis. Further data on TPE-free treatment protocols may help determine the contribution of antigen stimulation during TPE to the observed antibody rebound. 34 35

Anti-ADAMTS13 IgG levels peaked approximately 1 week after the last TPE session, but the rate of the subsequent decrease showed considerable variability ( Fig. 3 ). Importantly, post-TPE values, measured 1 week after the last TPE session—but not pre-TPE values—were strongly correlated with the time until anti-ADAMTS13 IgG negativity and with the time until sustained partial ADAMTS13 remission in caplacizumab-treated patients ( Fig. 4C ).

Early prediction of an increased risk of delayed ADAMTS13 activity normalization might be essential for optimizing immunosuppressive treatment during caplacizumab therapy. Therefore, we aimed to identify clinical or laboratory factors that could predict this risk.

The investigated demographic and clinical factors were not associated with increased risk for delayed ADAMTS13 normalization, defined here as a sustained partial ADAMTS13 remission achieved after more than 30 days from the first TPE session ( Table 3 ). More surprisingly, none of the treatment options used in iTTP were significant predictors. Our results regarding the virtually absent effect of rituximab therapy—even if it was applied early—are in line with those of Völker et al 21 and Izquierdo et al. 22 These counterintuitive results may in part be due to the delayed effect of rituximab, but may also in part be due to bias. As rituximab use was not uniform in any of the cohorts, there is a chance that patients with low antibody levels and a consequentially rapid clinical response tended not to be treated with rituximab. Thus, the above negative results do not indicate the lack of effect of rituximab on antibody levels, only that such a study arrangement is not appropriate for assessing the effects of therapeutic modalities on antibody levels.

Importantly, we have shown that the anti-ADAMTS13 IgG level measured a week after stopping TPE at the first clinical response (post-TPE) was a strong predictor of delayed ADAMTS13 normalization ( Table 3 and Supplementary Table S1 , available in the online version)—and the only predictor in our group of caplacizumab-treated patients. Caplacizumab-treated patients with anti-ADAMTS13 IgG levels over 110 U/mL had a delayed ADAMTS13 normalization, whereas patients with lower values did not.

Although this cut-off has to be reconsidered based on additional data in the future, our observations suggest that the determination of anti-ADAMTS13 IgG levels 1 week after the last TPE session can be utilized to identify caplacizumab-treated patients who are at increased risk of delayed ADAMTS13 normalization and thus for whom additional immunosuppressive therapy may be beneficial. This strategy would also help avoid unnecessary additional immunosuppression in patients with low risk of a delayed ADAMTS13 normalization.

Besides measuring the anti-ADAMTS13 IgG level post-TPE, we also recommend the regular (e.g., weekly) monitoring of this parameter throughout the caplacizumab treatment, as this is one of the few available markers (besides the quantitative determination of anti-ADAMTS13 inhibitors) that provides information about the efficacy of the applied immunosuppressive therapy in the setting of a normal platelet count and an undetectably low ADAMTS13 activity. Monitoring anti-ADAMTS13 IgG levels should also be considered for iTTP patients treated with TPE without caplacizumab, to aid in decisions regarding eventual additional immunosuppressive therapy.

It is important to note that our study is a single-center one. This may be a limitation, on the one hand, in part due to the relatively low number of patients included, but on the other hand, it minimizes the bias originating from different treatment protocols or different levels of expertise in multiple centers. To reduce bias resulting from changes in iTTP treatment protocols over time—other than the addition of caplacizumab—our historical control group consisted only of iTTP cases after 2017, when rituximab therapy was routinely being used.

It has to be noted that the method used in our study to determine the strength of functional ADAMTS13 inhibition—namely, to measure the ADAMTS13 activity of a patient sample mixed and incubated with a normal human pooled sample in a 1:1 ratio—is not able to distinguish between samples with strong inhibition. This may in part underlie the lack of association between strength of inhibition in post-TPE samples and delayed ADAMTS13 normalization. The issue could be circumvented by mixing the patient and normal sample in different ratios, based on which the exact strength of inhibition can be calculated. However, this approach would be more cumbersome and expensive in our setting compared to the measurement of anti-ADAMTS13 IgG; nevertheless, it might be the option of choice for other laboratories. Future studies are needed to investigate whether the exact ADAMTS13 inhibitor levels can be used to predict delayed ADAMTS13 normalization in iTTP.

Finally, we note that we used the standard sample dilutions recommended in the description of the anti-ADAMTS13 IgG ELISA kit, which may lead to under-estimation of extremely high anti-ADAMTS13 IgG levels. However, we believe that this does not limit the applicability of our data for the direct comparison of different patient samples and, more importantly, for tracking changes of the anti-ADAMTS13 IgG levels of a single patient. On the contrary, our data demonstrate that results acquired by a simple, standard ELISA procedure can be utilized to determine which patients are at higher risk for a relapse or for a delayed partial ADAMTS13 normalization.

## Conclusion

To conclude, our data provide a detailed longitudinal description of anti-ADAMTS13 IgG antibody levels in caplacizumab-treated and non-caplacizumab-treated iTTP patients, including the description of a characteristic post-TPE antibody rebound phenomenon and the prolonged persistence of these antibodies in certain patients. Our data support the utility of anti-ADAMTS13 IgG levels 1 week after the end of the initial TPE episode to identify caplacizumab-treated patients with a higher risk for prolonged persistence of anti-ADAMTS13 IgG antibodies and a delayed normalization of ADAMTS13 activity. The identification of these patients may in turn allow for applying additional immunosuppression selectively in these patients and thus may be an important step toward a more effective, safer, and individualized treatment of iTTP patients.

## Notes

**What is known about this topic?**

- The therapeutic anti-VWF nanobody caplacizumab proved to be effective in treating acute iTTP episodes, leading to a prompt clinical response, and thus allowing for an earlier termination of TPE.
- Delayed normalization of ADAMTS13 activity was reported in certain cohorts of caplacizumab-treated iTTP patients. Although such a delay was not observed in all studies, the time until ADAMTS13 activity normalization in caplacizumab-treated iTTP patients was found to be variable.
- Discontinuation of caplacizumab therapy before the—at least partial—normalization of ADAMTS13 activity confers a high risk of acute iTTP recurrence. Thus, the identification of patients with a higher risk for delayed ADAMTS13 activity normalization is important.

**What does this paper add?**

- After an initial decrease of anti-ADAMTS13 IgG levels during TPE, we observed a rebound after stopping TPE upon clinical response in all caplacizumab-treated iTTP patients in our cohort. Anti-ADAMTS13 IgG levels peaked around 1 week post-TPE.
- Importantly, the anti-ADAMTS13 IgG level measured 1 week after stopping TPE (post-TPE) was associated with the time to ADAMTS13 activity normalization, and was the only significant predictor of delayed ADAMTS13 activity normalization in both caplacizumab-treated and non-caplacizumab-treated iTTP patients.
- We propose that the anti-ADAMTS13 IgG levels should be monitored in caplacizumab-treated patients. High post-TPE anti-ADAMTS13 IgG levels may help identify patients who have an increased risk of delayed ADAMTS13 activity normalization and who would thus benefit from eventual additional immunosuppression.
