# Supplementary material for: High Anti-ADAMTS13 IgG Levels after Plasma Exchange Predict Delayed ADAMTS13 Normalization in Immune-Mediated Thrombotic Thrombocytopenic Purpura
Source: Thromb Haemost. 2025 Sep 2;126(6):646–60. doi: 10.1055/a-2685-8118 (PMC13288346; doi:10.1055/a-2685-8118)

Supplementary Material

High anti-ADAMTS13 IgG levels after plasma exchange predict delayed ADAMTS13 normalization in immune-mediated thrombotic thrombocytopenic purpura

Marienn Réti,[1] Andreea-Adela Icleanu,[2,3] Andrea Várkonyi,[1] Ágnes Király,[1] Luca Bogsch,[1] Zita Farkas,[1] Péter Reményi,[1] Zoltán Prohászka,[2,3] and György Sinkovits [2,3]

[1]Department of Hematology and Stem Cell Transplantation, Central Hospital of Southern Pest - Institute of Hematology and Infectious Diseases, Budapest, Hungary

[2]Department of Internal Medicine and Hematology, Semmelweis University, Budapest, Hungary

[3]Research Group for Immunology and Hematology, Semmelweis University - Hungarian Research Network (Office for Supported Research Groups), Budapest, Hungary

Treatment regime of immune-mediated thrombotic thrombocytopenic purpura (iTTP) patients

Establishing the clinical diagnosis

The clinical diagnosis of thrombotic thrombocytopenic purpura (TTP) was established when a combination of unexplained thrombocytopenia and Coombs-negative fragmentocytic haemolytic anaemia with or without ischemic organ dysfunction symptoms was noticed. The diagnosis was confirmed by detecting ADAMTS13 deficiency and the presence of an anti-ADAMTS13 inhibitor, which the Research Laboratory of Semmelweis University subsequently performed from a blood sample drawn before the therapy was started.

Therapeutic plasma exchange (TPE)

After establishing the clinical diagnosis and without waiting for the ADAMTS13 results, all patients received daily TPE, with 1–1.5 plasma volume was exchanged in each session, substituted with a combination of 5% human albumin and fresh frozen plasma (FFP), the latter given in the second half of the TPE procedures. TPE was stopped when the platelet

count reached >150 x 10^9/L without signs of haemolysis for at least 48 hours. Between 2 consecutive TPE procedures, 2-3 units of FFP were transfused in slow-rate infusion at the physician's discretion, which was continued for a few days after ending TPE treatment. In case of exacerbation, TPE was restarted.

Immunosuppression (ISU)

All patients received daily i.v. methylprednisolone (1-2 mg/kg/day), gradually tapered after a clinical response was achieved.

Rituximab (100 mg each week for 4 weeks) was offered to severe, refractory or relapsing patients with negative HBV/HCV status. It was started when the patients (or their legal representative) signed the informed consent, the off-label approval of the National Institute of Pharmacy was received, and the named patient-based financing approval request was submitted to the National Health Insurance Fund. Premedication with methylprednisolone (40-125 mg iv), chloropyramine (10-20 mg iv) and metamizole-sodium (1 g iv) was used to reduce allergic infusion-related reactions. If clinical symptoms permitted, TPE was withheld the next day following each infusion of rituximab and 2-4 units of FFP were transfused instead of TPE. Over the years, we have tried to start rituximab increasingly earlier, especially in cases receiving caplacizumab therapy as well.

Cyclophosphamide (500-1000 mg 1-2 times) and bortezomib (1.3 mg/m^2 each week for 3 weeks) were also added in some refractory cases or when significant antibody rebound was detected, while daratumumab (16 mg/kg each week, twice) was used instead of rituximab for a rituximab-intolerant patient. Off-label approvals of the National Institute of Pharmacy and named patient-based financing approval by the National Health Insurance Fund were also required for bortezomib and daratumumab treatments. ADAMTS13 activity of patients was regularly checked, and in case of a re-decrease of the ADAMTS13 activity below 30 %, the rituximab treatment mentioned above was used for relapse prevention.

Caplacizumab therapy

In 10 cases, caplacizumab was added to the standard of care (SOC; daily TPE + immunosuppression) when the drug was available. In all cases, caplacizumab treatment was started after having received the ADAMTS13 results. Sanofi’s Access Program provided caplacizumab in half of the cases. In the other half of cases, the hospital covered the cost of caplacizumab with or without later reimbursement. The treatment was given according to the drug’s instructions for use: The first 10 mg dose of caplacizumab was intravenously given

before the plasma exchange treatment, followed by 10 mg subcutaneous caplacizumab injections daily (given immediately after each plasma exchange) and continued daily for 30 days after discontinuation of plasma exchange treatments. Treatment could be extended for patients who have not yet normalised ADAMTS13 activity.

We were unable to continue caplacizumab treatment for patients not enrolled in Sanofi's Access Program after their discharge from the hospital due to financial constraints.

Other treatments

Low-dose aspirin and a prophylactic dose of LMWH were added to therapy when platelet count reached a stable level of > 50 x 10^9/L; both drugs were withheld during caplacizumab treatment. All patients received folate, potassium supplementation, gastric mucous membrane protection, and treatments of comorbidities if necessary. Red blood cell transfusions were given according to the Hungarian blood transfusion guidelines. Platelet transfusions were withheld except for some cases of central catheter insertion.

Outcome definitions (based on the 2021 consensus report of the International Working Group for TTP, with modifications) [23]

Initial platelet response: Platelet count ≥ 150 x 10^9/L for the first time.

Clinical response: Sustained (>2 consecutive days) platelet count ≥ 150 x 10^9/L and no signs of haemolysis allowing the discontinuation of TPE.

Permanent platelet response: Platelet count ≥ 150 x 10^9/L for at least 30 days.

Remission: Sustained clinical response with no TPE and no anti-VWF therapy for ≥ 30 days or until the first day of ADAMTS13 activity permanently above 20%, whichever occurs first.

Clinical exacerbation: Platelet count decreases to < 150 x 10^9/L (other causes of thrombocytopenia excluded) less than 30 days after an initial clinical response, with deficient ADAMTS13 activity, due to which TPE has to be restarted.

Sustained partial ADAMTS13 remission: ADAMTS13 activity ≥ 20% for at least 30 days (starting at least two days after the last TPE session and allowing single low values not lower than 17% (i.e. 85% of the threshold).

Supplementary Table S1: Results of univariable logistic regression models for estimating the risk of delayed ADAMTS13 normalization.

	**Odds ratio** per unit change (with 95% CI)	**p-value**
Age (years)	1.01 (0.96-1.06)	0.6613
Male sex	0.31 (0.05-1.87)	0.1864
Clinical relapse (vs. first episode)	0.44 (0.10-1.90)	0.2521
Platelet count before TPE start (10^9/L)	1.00 (0.92-1.09)	0.9737
Caplacizumab therapy	1.44 (0.30-6.99)	0.6394
Rituximab therapy	1.60 (0.32-8.03)	0.5514
Rituximab on the first week after TPE start	0.72 (0.17-2.98)	0.6413
TPE sessions until first clinical response (n)	1.01 (0.86-1.20)	0.8752
TPE sessions until clinical remission (n)	1.10 (0.96-1.25)	0.1710
Corticosteroid starting dose (mg)	0.98 (0.95-1.01)	0.1227
Corticosteroid starting dose per body mass (mg/kg)	1.50 (0.45-5.03)	0.4912
Other immunosuppressive therapy	1.82 (0.29-11.27)	0.5059
Post-TPE ADAMTS13 activity (%)	0.92 (0.80-1.04)	0.1626
Post-TPE ADAMTS13 inhibitor (activity of the sample mixed 1:1 with normal human sample, %)	1.03 (1.00-1.06)	0.0513
Pre-TPE anti-ADAMTS13 IgG (IU/mL)	1.026 (1.004-1.048)	0.0156
Post-TPE anti-ADAMTS13 IgG (IU/mL)	1.032 (1.010-1.054)	**0.0030**

Delayed ADAMTS13 normalization: Sustained partial ADAMTS13 remission is achieved after more than 30 days from the first TPE session. Sustained partial ADAMTS13 remission:

ADAMTS13 activity ≥ 20% for at least 30 days (allowing single low values not lower than 17% (85% of the threshold) and starting at least two days after the last TPE session).

Pre-TPE samples were taken directly before the initiation of TPE or on the day before in two control patients. Post-TPE samples were taken approximately one week (median 6, IQR: 5-8.5 days) after the end of the first TPE series (discontinued after reaching the first clinical response), and before the second TPE series in exacerbating control patients.

Other immunosuppressive agents applied: cyclophosphamide, bortezomib, daratumumab.

Significant p-values after the Bonferroni correction (significance limit p= 0.0033) are indicated in bold.

Supplementary Fig. S1. Overview of patient inclusion and clinical outcomes. The diagnosis of acute iTTP was based on the following criteria: (1) clinical diagnosis: unexplained thrombocytopenia (platelet count below 150 x 10^9/L) and microangiopathic hemolytic anemia (Coombs-negative hemolytic anemia, elevated LDH, schistocytes on the blood smear); (2) deficient ADAMTS13 activity (<10%, measured by a FRETS-VWF73 assay, as described below); (3) detectable anti-ADAMTS13 autoantibodies (by a functional inhibitory assay and/or an anti-ADAMTS13 IgG ELISA). There were 52 acute iTTP episodes in the inclusion period. The treatment regimen included caplacizumab for all acute episodes as long as it was available. Caplacizumab therapy had to be discontinued for one patient after 19 days due to the patient's decision; this patient was excluded selectively from the analyses concerning outcome, clinical or laboratory data after the discontinuation. Out of the 42 non-caplacizumab-treated (control) iTTP episodes, four cases reaching stable clinical response were transferred to other hospitals, whereas one patient died within one day; these cases were not included in the study. Six control patients did not have positive anti-ADAMTS13 IgG values at the onset of one of their acute episodes; these episodes were excluded from the control group. Further three control patients had multiple control episodes: for these patients, the earliest of the episodes was included, whereas other episodes were excluded. Two patients had both caplacizumab-treated and non-caplacizumab-treated episodes.

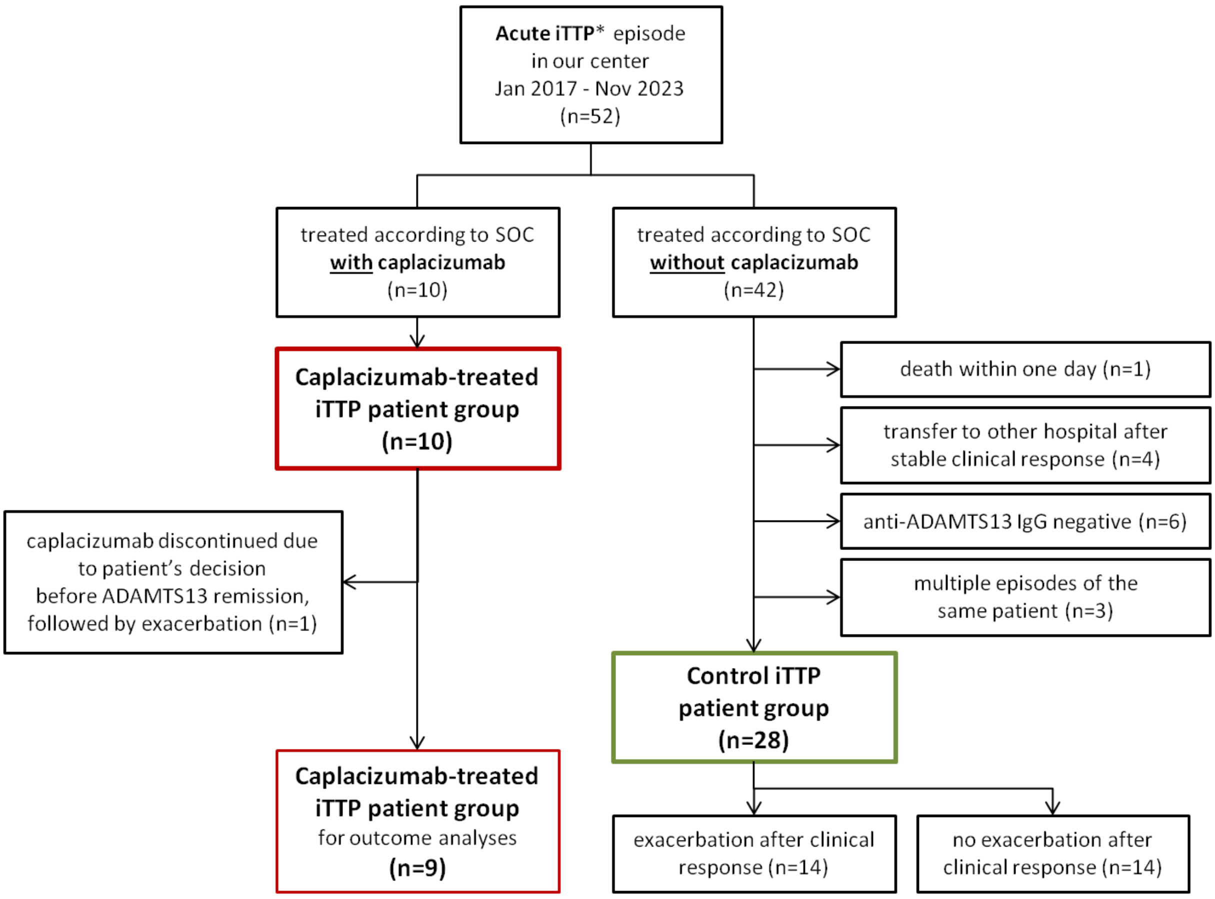
Acute iTTP* episode
in our center
Jan 2017 - Nov 2023
(n=52)
treated according to SOC
with caplacizumab
(n=10)
treated according to SOC
without caplacizumab
(n=42)
Caplacizumab-treated
iTTP patient group
(n=10)
death within one day (n=1)
transfer to other hospital after
stable clinical response (n=4)
anti-ADAMTS13 IgG negative (n=6)
caplacizumab discontinued due
to patient's decision
before ADAMTS13 remission,
followed by exacerbation (n=1)
multiple episodes of the
same patient (n=3)
Control iTTP
patient group
(n=28)
Caplacizumab-treated
iTTP patient group
for outcome analyses
(n=9)
exacerbation after clinical
response (n=14)
no exacerbation after
clinical response (n=14)

Supplementary Fig. S2. ADAMTS13 activity values of exacerbating and non-exacerbating control iTTP patients. Results of iTTP patients treated according to the standard of care but without caplacizumab therapy (control patients) are shown. Half (14/28) of these patients had an exacerbation following the first TPE series, and thus a second TPE series had to be performed in their cases.

Individual measurements of exacerbating and non-exacerbating control patients are plotted on panels **A** and **B**, respectively. Subsequent values of the same patient are connected with a line. The line between two points is bold and colored green (first TPE series) or blue (second TPE series) if TPE sessions were performed in the period between the two sampling times, within 3 days of the latter sampling time. The start of sustained (for at least 30 days) partial ADAMTS13 remission (activity >20%) is marked by yellow rhombi.

Mean ADAMTS13 activity values and their 95% confidence intervals are shown on panel **C**. For each day, the next ADAMTS13 value (on the same day or thereafter) was considered for each patient. Samples taken more than a week (until day 30) or two weeks (from day 30) after the given time point were excluded from the analysis. Results of caplacizumab-treated patients are shown as a comparison.

The vertical line indicates the zero time point, which is the last day of the first TPE series. The horizontal dotted line indicates the threshold of partial ADAMTS13 remission (20%).

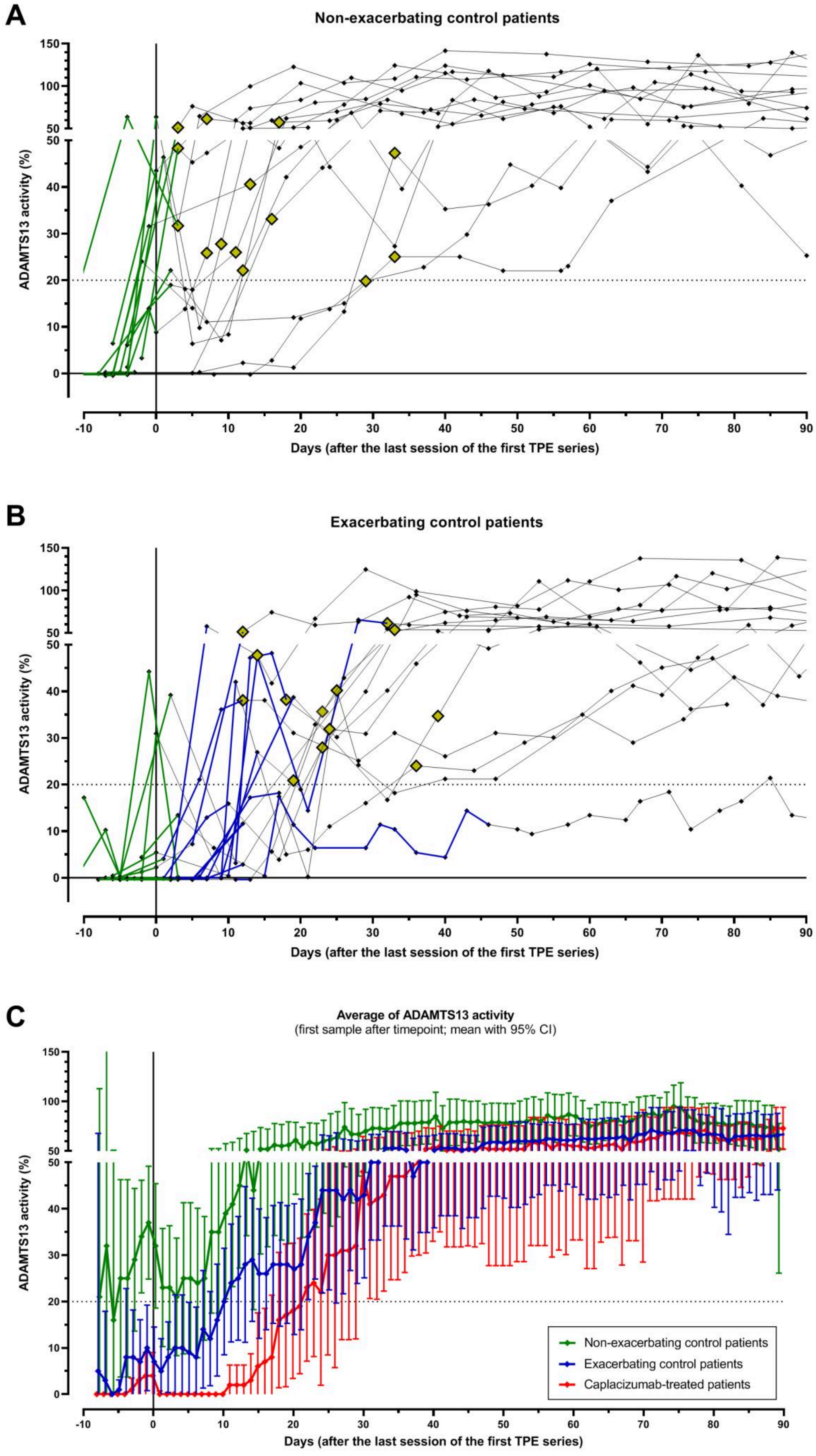
A
Non-exacerbating control patients
ADAMTS13 activity (%)
Days (after the last session of the first TPE series)
B
Exacerbating control patients
ADAMTS13 activity (%)
Days (after the last session of the first TPE series)
C
Average of ADAMTS13 activity
(first sample after timepoint; mean with 95% CI)
ADAMTS13 activity (%)
Non-exacerbating control patients
Exacerbating control patients
Caplacizumab-treated patients
Days (after the last session of the first TPE series)

Supplementary Fig. S3. ROC (receiver operating characteristic) analysis of post-TPE anti-ADAMTS13 IgG values to distinguish between patients with or without a delayed ADAMTS13 normalization. ADAMTS13 normalization is considered delayed if the sustained partial ADAMTS13 remission (ADAMTS13 activity over 20% for at least 30 days) is achieved after more than 30 days from the first TPE session. Post-TPE samples were taken 6 (IQR: 5-8) days after the last TPE session of the first TPE series. Results of caplacizumab-treated patients (A) and iTTP patients not treated with caplacizumab (B) were analyzed separately.

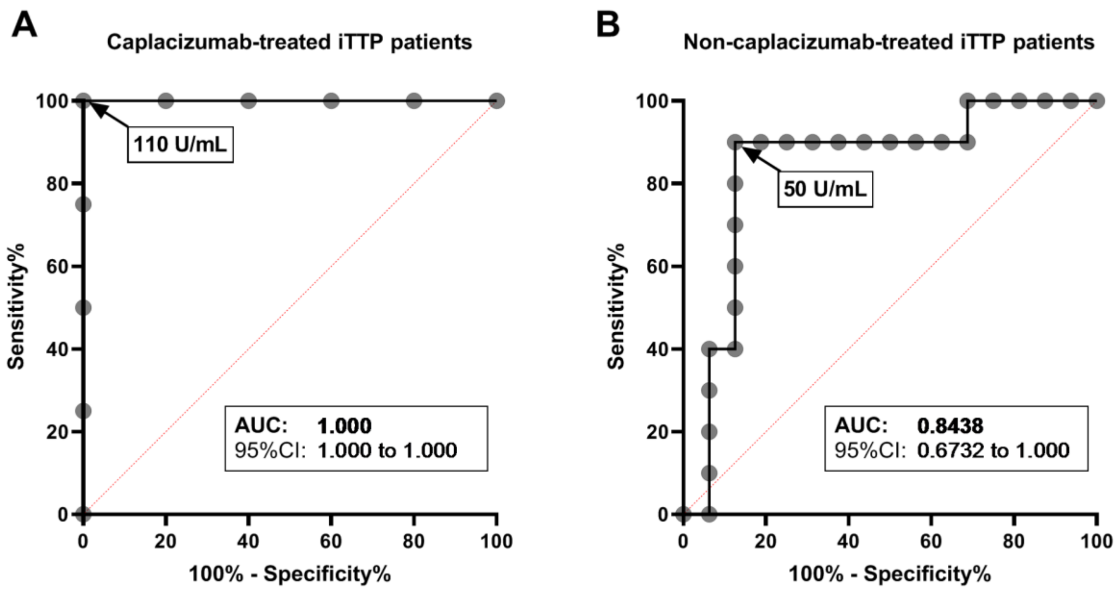

Supplement: Supplementary file 1 — Supplementary Material [file 10-1055-a-2685-8118-s25030128.pdf]
